# Treatment planning of volumetric modulated arc therapy and positioning optimization for hippocampal‐avoidance prophylactic cranial irradiation

**DOI:** 10.1002/acm2.13217

**Published:** 2021-04-07

**Authors:** Qi Fu, Deqi Chen, Hui Yan, Jiayun Chen, Ji Zhu, Lingling Yan, Yingjie Xu, Lei Deng, Kuo Men, Jianrong Dai

**Affiliations:** ^1^ Department of Radiation Oncology National Cancer Center/Cancer Hospital Chinese Academy of Medical Science Peking Union Medical College Beijing China

**Keywords:** prophylactic cranial irradiation, hippocampus, volumetric modulated arc therapy, helical tomotherapy

## Abstract

**Background:**

Hippocampal‐avoidance prophylactic cranial irradiation (HA‐PCI) offers potential neurocognitive benefits but raises technical challenges to treatment planning. This study aims to improve the conventional planning method using volumetric modulated arc therapy (VMAT) technique and investigate a better patient’s head positioning to achieve a high quality of HA‐PCI treatment plans.

**Methods:**

The improved planning method set a wide expansion of hippocampus as a special region for dose decline. The whole brain target was divided into two parts according to whether the slice included hippocampus and their optimization objectives were set separately. Four coplanar full arcs with partial field sizes were employed to deliver radiation dose to different parts of the target. The collimator angle for all arcs was 90°. Tilting patient’s head was achieved by rotating CT images. The improved planning method and tilted head positioning were verified using datasets from 16 patients previously treated with HA‐PCI using helical tomotherapy (HT).

**Results:**

For the improved VMAT plans, the max and mean doses to hippocampus were 7.88 Gy and 6.32 Gy, respectively, significantly lower than those for the conventional VMAT plans (*P* < 0.001). Meanwhile, the improved planning method significantly improved the plan quality. Compared to the HT plans, the improved VMAT plans result in similar mean dose to hippocampus (*P* > 0.1) but lower max dose (*P* < 0.02). Besides, the target coverage was the highest for the improved VMAT plans. The tilted head positioning further reduced the max and mean doses to hippocampus (*P* < 0.05), significantly decreased the max dose to lens (*P* < 0.001) and resulted in higher plan quality as compared to nontilted head positioning.

**Conclusions:**

The improved planning method enables the VMAT plans to meet the clinical requirements of HA‐PCI treatment with high plan quality and convenience. The tilted head positioning provides superior dosimetric advantages over the nontilted head positioning, which is recommended for clinical application.

## 1 INTRODUCTION

Prophylactic cranial irradiation (PCI) is an effective way to prevent brain metastases (BM) in lung cancer patients.^1^, ^2^, ^3^, ^4^ Several clinical trials have shown that PCI significantly decreased the incidence of brain metastases compared with observation.^5^, ^6^, ^7^ However, the use of PCI will induce adverse effects like neurocognitive deficits, which are believed to be caused by radiation induced damage of neural stem cell (NSC) compartment in the hippocampus.^8^, ^9^ In order to reduce these cognitive side‐effects, it is necessary to minimize radiation dose to the hippocampus during PCI. That is to perform hippocampal‐avoidance PCI (HA‐PCI).

Among the current radiotherapy techniques, helical tomotherapy (HT) is considered to be the preferred technique to treat complex treatment situations because the radiotherapy modality of helical tomoscan has a powerful modulation capability. Many studies have confirmed that using HT technique could achieve superior dose conformity and homogeneity for concave or even hollow target adjacent to sensitive structures, which is just right for the HA‐PCI treatment.^10^, ^11^ However, due to its high cost, HT technique is not common or even unavailable in most hospitals and clinics of China. For instance, a total of 166 patients received PCI at Cancer Hospital Chinese Academy of Medical Science in 2018, only 18% of who were treated with HA‐PCI using HT. The rest were all treated with PCI without avoiding hippocampus using traditional conformal radiotherapy (CRT). Compared with HT, volumetric modulated arc therapy (VMAT) is a far more common technique. But most studies showed that it is usually less than satisfactory in hippocampus sparing and dose homogeneity of target.^12^, ^13^, ^14^ Therefore, HA‐PCI using a VMAT is not readily available in the clinic. In this study, we aimed to improve the planning method of conventional VMAT plans to achieve a high plan quality comparable to that of HT plans. Besides, we attempted to change patient’s head positioning to further improve the plan quality for HA‐PCI.

## 2 METHODS AND MATERIALS

### 2.1 Patients selection and contouring

**Fig. 1:**
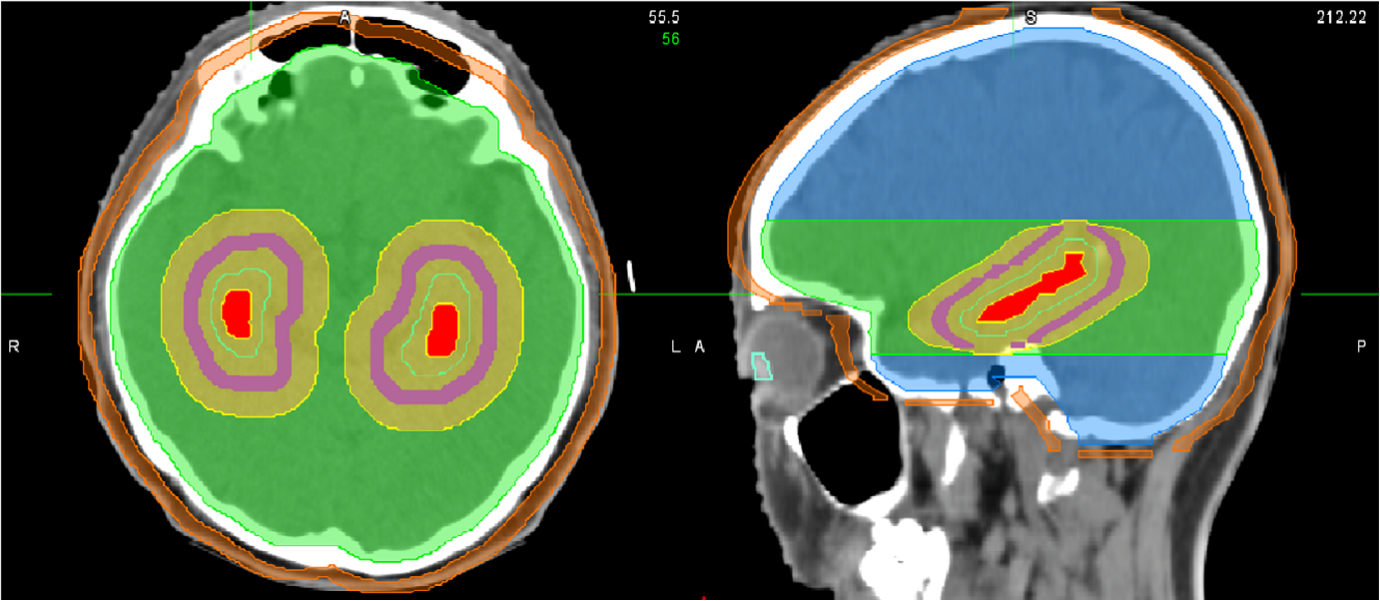
Axial and sagittal CT images showing hippocampus (red), hippocampus PRV (cyan line), PTV_plan1 (green), PTV_plan2 (blue), dose decline region (yellow), ring PRV (pink), and ring PTV (orange).

For this planning study, 16 patients, who had been previously treated with HA‐PCI using HT in 2018–2019, were randomly selected. All patients had undergone cranial computed tomography (CT) and magnetic resonance imaging (MRI) scans, both with 2‐mm thickness. These images were fused in Pinnacle v9.10 (Philips Radiation Oncology Systems, Fitchburg, WI, USA) for contouring and planning. The hippocampus was contoured on T1‐weighted MRI axial sequences following RTOG 0933 protocol. For the 16 patients, the maximum, minimum and mean volumes of hippocampus were 7.19 cc, 3.82 cc and 5.38 cc, respectively. A hippocampal Planning Risk Volume (PRV) was defined as the hippocampus plus 5‐mm uniform expansion.^15^ The planning target volume (PTV) was a 3‐mm uniform expansion of the whole brain excluding the hippocampal PRV. Two dose‐shaping structures are created: ring PRV was from 3 to 8 mm outside of the hippocampus PRV, and ring PTV was from 5 to 10 mm outside of the PTV. These structures were showed in Fig. 1. Additionally, normal tissue structures, including the lens, optic nerve, optic chiasm, brainstem, were contoured for dose evaluation.

### 2.2 Treatment planning

Volumetric modulated arc therapy plans for each patient were generated in Pinnacle v9.10, separately using the conventional and improved planning methods. For dose calculation, the Adaptive Convolve algorithm with heterogeneity correction was used, with a dose grid resolution of 2 mm. Treatment was delivered using a Elekta VersaHD^TM^ (Elekta, Crawley, United Kingdom), 120‐leaf MLC, and 6‐MV photon beams with a maximum dose rate of 700 MU/min. Maximum leaf motion was limited to 1 cm/deg (6 cm/s). Gantry spacing was set to 3°. For the present study, the clinically used HT plans were included in comparison in order to verify that the improved VMAT plans can be applied in clinic. All the HT plans were generated using a 2.5‐cm field width and dynamic jaws. The pitch was selected as 0.257 and the modulation factor was set between 2.0 and 3.0. The treatment prescription to the whole brain PTV was set to deliver 25 Gy in 10 fractions, with at least 90% of the PTV receiving 100% of the prescription dose (PD). The max and mean dose to the hippocampus were limited to 9 Gy and 7 Gy respectively. The max dose to lens could not exceed 8 Gy.

### 2.3 Improved VMAT planning method

**Fig. 2:**
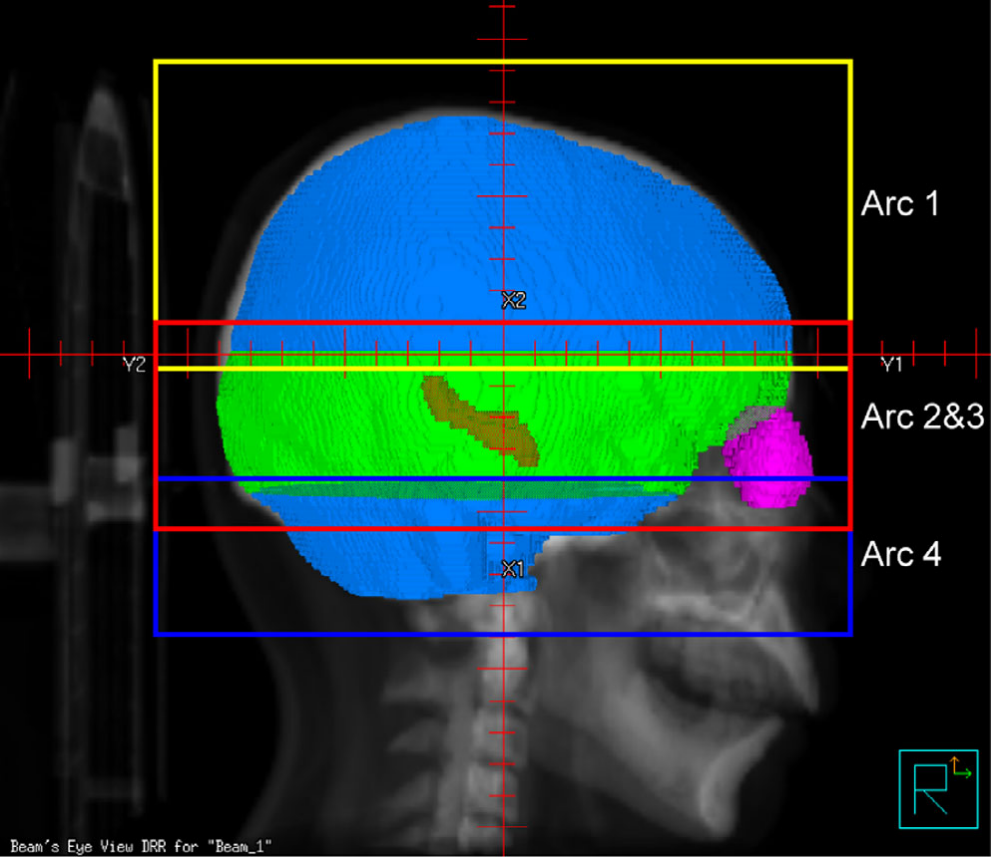
Beam's eye view (BEV) at 270° showing field sizes of Arc 1 (yellow), Arc 2 and 3 (red), and Arc 4 (blue).

The conventional VMAT plans in this study employed double full coplanar arcs with a collimator of 0°. The main optimization objectives were shown in Table 1. On the base of this conventional planning method, the improved planning method was improved in several ways:

1. Setting a special region for dose decline. In the whole brain PTV, it takes a certain spatial distance to drop from the PD to the dose constraint of the hippocampus. In order to allow the optimizer to fulfill all the objectives more easily, we defined a dose decline region as the hippocampus expanded by 2 cm in left‐right and anterior‐posterior directions and 1 cm in superior‐inferior direction (the yellow region showed in Fig. 1). Then we defined the PTV_plan as the whole brain PTV subtracting the dose decline region. It was used only for the improved VMAT plan optimization and required to achieve the PD coverage as high as possible. In addition, a ring PRV was set in the dose decline region to help control the dose to hippocampus. The main optimization objectives used for the improved VMAT plans were listed in Table 1. Note that we tightened the hippocampal dose constraints to seek a lower hippocampal dose without sacrificing the PTV coverage.
2. Dividing the whole brain target into two parts. Inhomogeneous dose distribution can easily occur in the slices including the hippocampus, especially near the hippocampus. To control hot spots and cold spots more efficiently, we divided the PTV_plan into two parts: the PTV_plan in the slices that contains the hippocampal dose decline region was defined as PTV_plan1, and the rest was PTV_plan2 (see Fig. 1). Therefore, optimization objectives could be set separately for these two targets. As shown in Table 1, the weights of the objectives were higher and Max Dose goal was stricter for PTV_plan1 than those for PTV_plan2.
3. Using four coplanar full arcs with limited field sizes. The large size of whole brain target usually requires a large irradiation field, leading to a large range of MLC leaf motion. This may affect the target exposure and hippocampus sparing. By limiting jaw opening size, we achieved that the upper and lower PTV_plan1 were irradiated separately using two coplanar full arcs (Arc 1 and 4) and the PTV_plan2 was irradiated using a pair of coplanar full arcs (Arc 2 and 3). As shown in Fig. 2, the fields of adjacent arcs overlapped each other by approximately 1.5 cm.
4. Rotating collimator to 90° for all arcs. The collimator angle of 90° is better for sparing bilateral structures within a target, such as hippocampi, eyeballs, and lenses.

**Table 1:** Main optimization objectives of the improved VMAT plan.

Structure	Objective	Conventional plan		Improved plan	
		Value	Weight	Value	Weight
PTV	Max Dose	26 Gy	100	–	–
	Min Dose	24.5 Gy	80	–	–
	Min DVH	25 Gy, 95%	100	25 Gy, 95%	100
	Uniform Dose	25.5 Gy	20	–	–
PTV_plan1^a^	Max Dose	–	–	26 Gy	100
	Min Dose	–	–	24.5 Gy	80
	Min DVH	–	–	25 Gy, 100%	100
	Uniform Dose	–	–	25.5 Gy	100
PTV_plan2^a^	Max Dose	–	–	27.5 Gy	60
	Min Dose	–	–	24.5 Gy	70
	Min DVH	–	–	25 Gy, 100%	70
	Uniform Dose	–	–	25.5 Gy	100
Hip L	Max Dose	10 Gy	50	6 Gy	50
Hip R	Max Dose	10 Gy	50	6 Gy	50
Hip PRV	Max DVH	13 Gy, 1%	30	8 Gy, 1%	30
	Max EUD	8 Gy	1	5 Gy	1
Lens L	Max Dose	7 Gy	10	7 Gy	10
Lens R	Max Dose	7 Gy	10	7 Gy	10
Ring PRV	Max Dose	–	–	18 Gy	10
Ring PTV	Max Dose	24 Gy	20	24 Gy	20

VMAT, volumetric modulated arc therapy; PTV, planning target volume.

^a^ PTV_plan is PTV_plan1 plus PTV_plan2.

### 2.4 Tilted head positioning

**Fig. 3:**
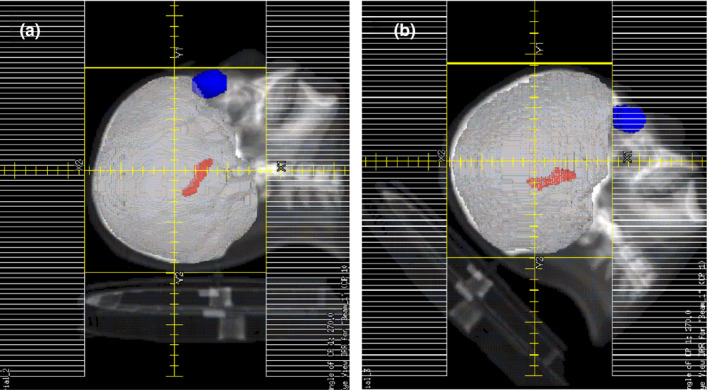
BEV at 270° for nontilted (a) and tilted (b) head positionings, with a collimator angle of 90°. PTV is shown in gray, hippocampus in red, eyeballs in blue.

Hippocampus is located in the lower part of temporal lobe. As can be seen in Fig. 3, its long axis appears tilted in the sagittal plane. Thus, if a patient’s head is tilted forward at a certain degree, the long axis of the hippocampus will be turned to be parallel to gantry rotation axis, which may help multi‐leaf collimator (MLC) leaves to spare the hippocampus. Two recent studies reported that when patients received HA‐PCI with 30° tilted head positioning, the dose to the hippocampus and other normal tissues could be further decreased.^16^, ^17^ In order to spare normal tissues, especially the hippocampus, to the maximum, we chose the tilt angle of patient’s head to be 45° for this study after analyzing hippocampal tilt angle and eyeballs position relative to brain for different patients. For the sake of simplicity, we rotated CT images of 0° position to simulate the situation of a 45° tilted head position. The original CT images were imported to Image J software, rotated along the left‐right axis by 45°, and then resampled to create the rotated new CT images. The contours in the 0° CT images were then mapped to the rotated CT images after fusing the two datasets together using rigid registration.

### 2.5 Plan evaluation

The dosimetric parameters recommended by RTOG 0933 protocol were extracted from the dose–volume histogram (DVH) and analyzed for each plan, which include D2%, D50%, D98% (dose to 2%, 50% and 98% of the PTV), V_PTV,ref_ (the volume of PTV that receives dose equal to or greater than PD), V_ref_ (the volume receiving the PD). For the PTV, homogeneity index (HI) defined as (D2%‐D98%)/D50% and conformal index (CI) defined as V_PTV,ref_^2^/(V_PTV_ × V_ref_) were evaluated. For the organs at risk (OARs), the max and mean dose to hippocampus and the max dose to lens were evaluated. Additionally, the number of monitor units (MU) was recorded and evaluated. Statistical comparisons between different plans were performed using the two‐sided paired t‐test at 5% level significance.

## 3 RESULTS

### 3.1 Improved VMAT vs. conventional VMAT and HT

The dose parameters for the improved VMAT plans, conventional VMAT plans and HT plans used in clinic were compared in Table 2. The range of mean hippocampal dose was 23.0%~32.5% of the PD for the improved VMAT plans, 33.4%~39.2% for the conventional VMAT plans, and 21.2%~34.6% for the HT plans. Among the three plans, the improved VMAT plans had the highest PD coverages of the PTV, PTV_plan and PTV‐15mm. Compared to the conventional VMAT plans, the improved VMAT plans resulted in an approximately 30% reduction in both max and mean doses to hippocampus. No obvious differences in the mean hippocampal dose were found between the improved VMAT plans and the HT plans, but the max hippocampal dose was significantly lower for the improved VMAT plans (*P* < 0.02). In addition, no significant variations were observed with the max dose to lens among the three plans.

**Table 2:** Comparison of dose parameters among the HT plans, conventional VMAT plans, and improved VMAT plans with nontilted head positioning and tilted head positioning in 16 patients.

Structure	Dose parameter	HT	VMAT			P_13_	P_23_	P_34_
			Conventional	Improved	Tilted			
PTV	V_25 Gy_ (%)	90.41 ± 1.04	90.72 ± 0.76	91.20 ± 0.83	91.04 ± 0.73	0.007	0.011	0.263
PTV_plan	V_25 Gy_ (%)	96.41 ± 1.21	95.65 ± 0.81	98.47 ± 0.84	99.54 ± 0.20	<0.001	<0.001	<0.001
PTV‐15mm^a^	V_25 Gy_ (%)	97.22 ± 1.16	95.45 ± 0.76	98.62 ± 0.74	99.07 ± 0.37	0.001	<0.001	0.008
Hip L	D_max_ (Gy)	8.81 ± 0.96	10.97 ± 0.31	7.90 ± 1.01	7.23 ± 0.28	0.013	<0.001	0.021
	D_mean_ (Gy)	6.67 ± 0.92	8.88 ± 0.32	6.35 ± 0.70	5.84 ± 0.25	0.225	<0.001	0.008
Hip R	D_max_ (Gy)	9.10 ± 1.07	11.06 ± 0.36	7.85 ± 0.84	7.18 ± 0.33	0.004	<0.001	0.007
	D_mean_ (Gy)	6.70 ± 0.91	9.08 ± 0.34	6.29 ± 0.60	5.91 ± 0.28	0.149	<0.001	0.012
Lens L	D_max_ (Gy)	6.74 ± 1.12	6.96 ± 0.59	7.19 ± 0.68	4.67 ± 1.18	0.088	0.265	<0.001
Lens R	D_max_ (Gy)	6.85 ± 1.16	7.05 ± 0.51	7.27 ± 0.55	4.27 ± 1.14	0.170	0.270	<0.001

HT, helical tomotherapy; VMAT, volumetric modulated arc therapy; PTV, planning target volume.

^a^ PTV‐15mm is defined as the PTV subtracting 15‐mm uniform expansion of hippocampus.

### 3.2 Tilt vs. nontilt

**Fig. 4:**
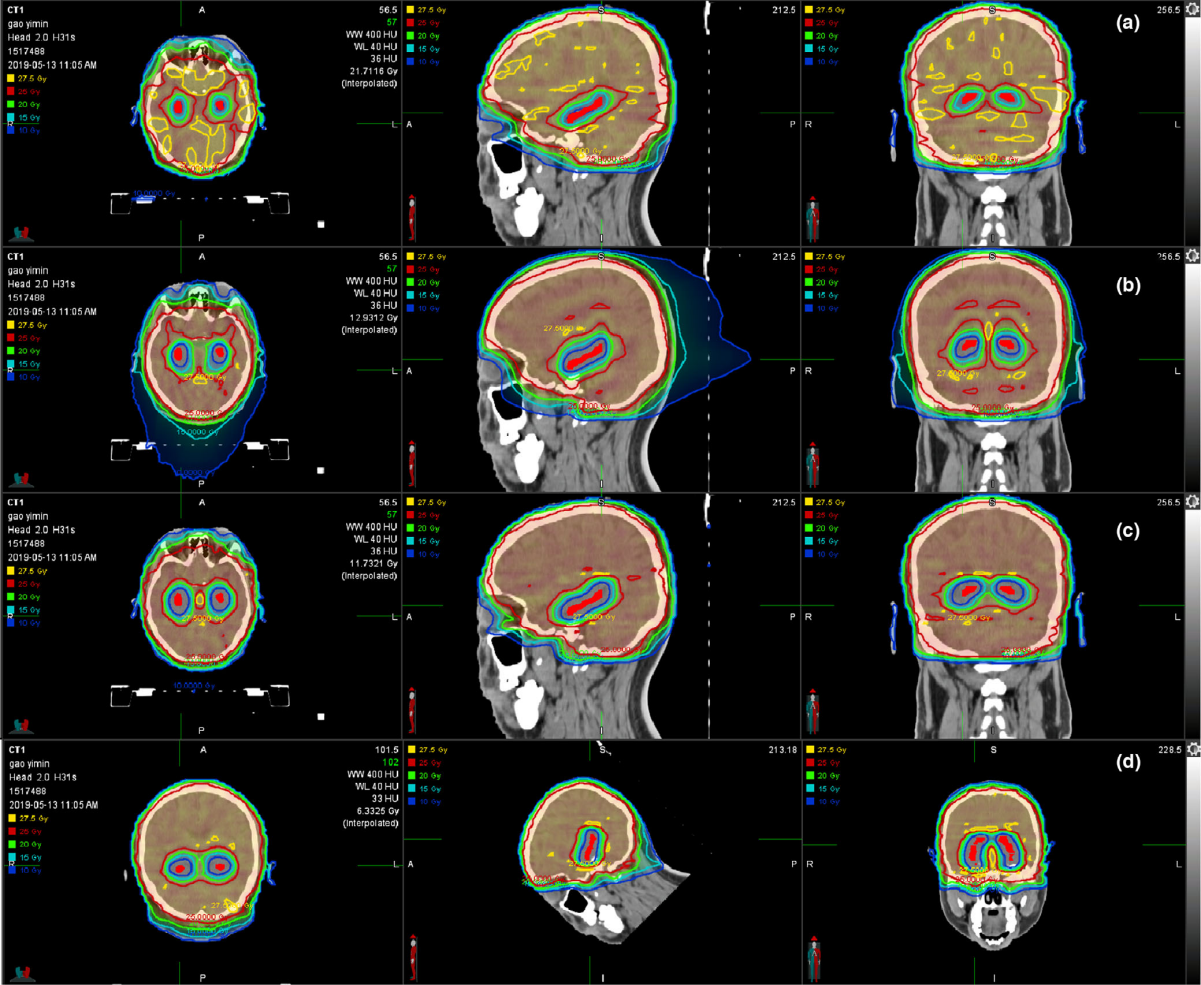
Dose distributions of the conventional VMAT plans (a), the HT plans (b), and the improved VMAT plans with nontilted positioning (c) and tilted head positioning (d). Hippocampus is shown in red.

The VMAT plans with tilted and nontilted head positioning were all generated using the improved planning method with the same optimization objectives. As shown in Table 2, when the patients tilted their head forward, the doses to the hippocampus and lens were all significantly decreased (*P* ≤ 0.021) while the coverage of the PTV_plan and PTV‐15mm were increased (*P* < 0.01). It is because tilting patient’s head forward leads to a decrease in the area of the hippocampus in axial plane (see Fig. 4) and makes MLC leaves spare the hippocampus more easily (as mentioned earlier), which all help reduce the dose to hippocampus. Moreover, when the patients tilted head forward, the lenses are blocked by collimator jaw at most beam angles so that the dose to lens is also significantly decreased (*P* < 0.001).

**Fig. 5:**
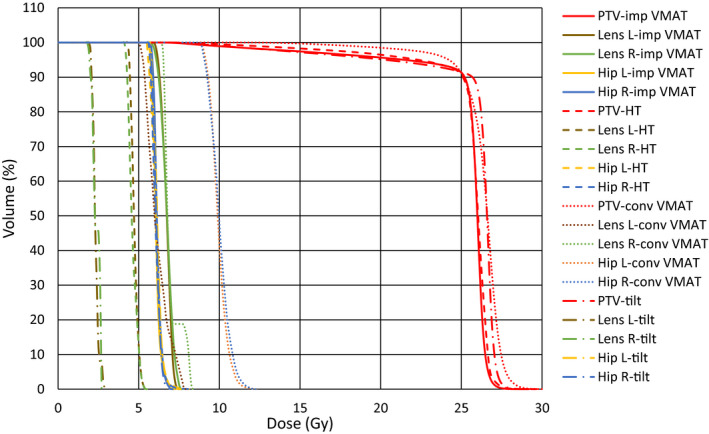
Dose–volume histogram of the conventional VMAT plans (a), the HT plans (b), and the improved VMAT plans with nontilted positioning (c) and tilted head positioning (d).

The CI, HI, and MU for the four plans were compared in Table 3. The improved VMAT plans resulted in the highest CI and lowest HI compared with the HT plans and the conventional VMAT plans, which indicated that the improved planning method could produce significantly high plan quality. However, the mean MU was significantly increased for the improved VMAT plans due to the increased number of beam arcs and modulation capability. Tilting patient’s head significantly decreased HI and MU (*P* < 0.001) while slightly decreased CI (*P* = 0.01), indicating that the plan quality was further improved. Figures 4 and 5 intuitively presented the significant differences in DVH and dose distribution among the four plans.

**Table 3:** Comparison of CI, HI and MU among the HT plans, conventional VMAT plans, and improved VMAT plans with nontilted head positioning and tilted head positioning in 16 patients.

Item	HT	VMAT			P_13_	P_23_	P_34_
		Conventional	Improved	Tilted			
CI	0.824 ± 0.141	0.816 ± 0.019	0.839 ± 0.030	0.822 ± 0.018	0.318	0.009	0.011
HI	0.115 ± 0.044	0.150 ± 0.014	0.084 ± 0.011	0.067 ± 0.010	<0.001	<0.001	<0.001
MU	–	1395.1 ± 140.7	2202.5 ± 159.1	1776.9 ± 98.5	–	<0.001	<0.001

HT, helical tomotherapy; VMAT, volumetric modulated arc therapy.

## 4 DISCUSSION

Prophylactic cranial irradiation plays an important role in the prevention of BM for lung cancer patients. But the resulting irradiation damage to NSC in the hippocampus may impact quality of life for patients. Therefore, RTOG 0933 and NRG CC001 suggested that hippocampus should be avoided during WBRT and PCI. Comparisons showed that hippocampal‐avoidance WBRT could obviously reduce neurocognitive dysfunction.^8^, ^9^ By using HT technique, Gutierrez and Gondi successively achieved HA‐WBRT,^12^, ^14^ and Marsh achieved HA‐PCI with the max hippocampal dose of 50% PD.^10^, ^11^ However, HT is not common for various reasons, while using the more common VMAT technique is hard to reach the strict dose constraint of hippocampus. Moreover, it is also lack of effective VMAT planning methods for reference. Thus, up to now very few hospitals and clinics treat patients with HA‐PCI treatment using VMAT.

Recently, Yuen et al have decreased the max hippocampal dose to 44% PD for HA‐WBRT using complex VMAT techniques, such as split‐arc and partial field.^18^ In the present study, we employed four coplanar full arcs and reduced the max hippocampal dose to 35% of the PD (even lower than the HT plans) while maintaining a high plan quality. The planning method we used is mainly improved from the following aspects.

Firstly, adequate space around the hippocampus was reserved for dose decline. Due to the strict hippocampal dose constraint, the low dose region is mainly concentrated around the hippocampus. In certain cases, the dose gradient is basically unchanged. Therefore, the difference between the max hippocampal dose constraint and the PD determines the space size required for dose decline as well as the PD coverage of the target. A lack of space may increase the dose to hippocampus and lead to heterogeneous dose distribution in the target. In this study, the dose decline from the PD (25 Gy) to the max hippocampus dose (9 Gy) needs approximately a 20‐mm expansion of the hippocampus. If the PD covers nearly 100% volume of the remaining PTV (PTV_plan), the coverage of the whole brain PTV can easily meet the treatment prescription (V_25 Gy_ ≥ 90%). Related studies have shown that the metastatic involvement of the NSC regions (especially hippocampus) is unusual and the risk for the metastases within 15 mm of the hippocampus is lower than 15%.^19^, ^20^ According to statistics, only 2.7% of the metastases appeared within 15 mm of the hippocampus due to inadequate dosage after HA‐PCI treatment.^21^ Although the present study reserved a larger region (than 15‐mm hippocampal expansion) for dose decline, the PTV_plan (PTV_plan1 + PTV_plan2) has been optimized to achieve a maximum PD coverage. This made low dose regions more concentrated in the low‐risk region of BMs around the hippocampus, which helps reduce not only the overall risk of BMs but also the dose to hippocampus. Table 2 has shown that the PD coverage of PTV‐15mm was significantly higher for the improved VMAT plans than the other two plans. Thus, we believe the risk of BMs is acceptable.

Secondly, the dose homogeneity of the target was further improved by dividing the target and then exposing different parts using different arcs with partial fields. Separately setting optimization objectives for the divided targets (PTV_plan1 and PTV_plan2) could realize more targeted control of unsatisfied dose distribution. We found that repeatedly using Uniform Dose objective to target for more times of optimization could decrease the HI by 30% at most while keeping the max hippocampal dose change within 5%. Besides, limiting field sizes of beam arcs to cover different parts of the targets could narrow the range of MLC leaf motion and increase modulation capability so as to allow the MLC to spare hippocampus without affecting target exposure.

**Fig. 6:**
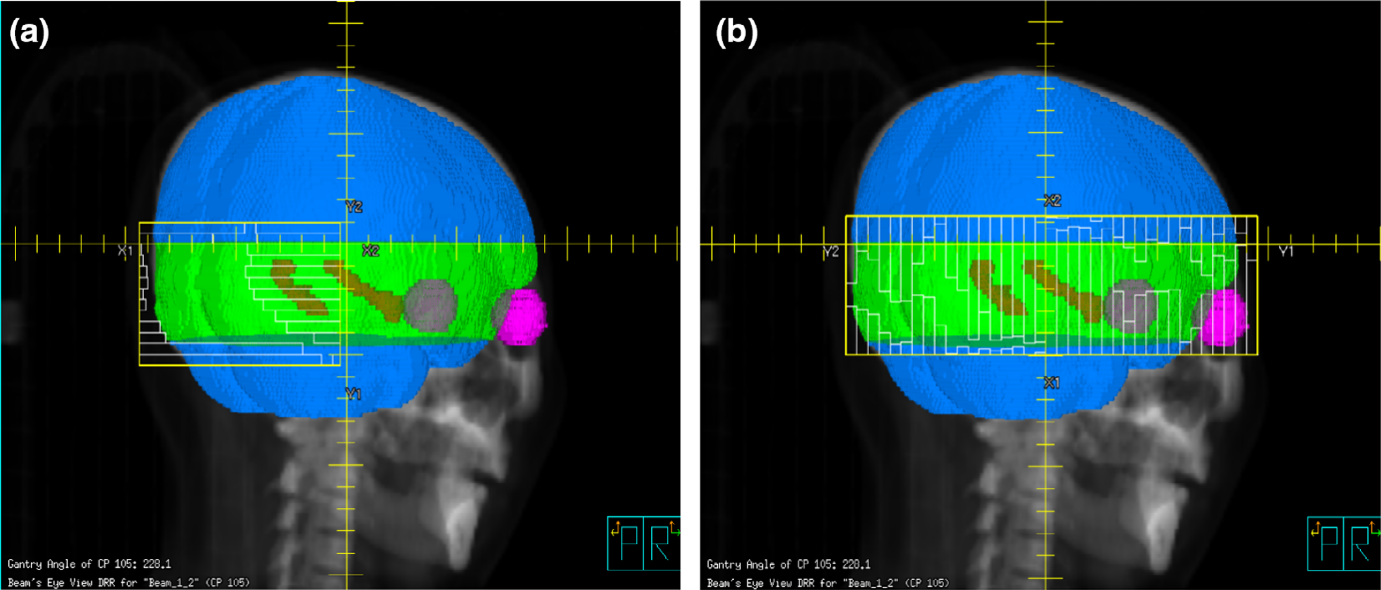
Examples of BEV with collimator angles of 0° (a) and 90° (b). PTV_plan1 is shown in green, PTV_plan2 in blue, hippocampus in red, eyeballs in purple.

Thirdly, the collimator angle was set to 90°, different from a small angle (5°–30°) applied in most studies. As shown in Fig. 6(a), when the collimator at 0° (or a small angle), some MLC leaf pairs need to both expose the target and spare two or more OARs simultaneously (such as left and right hippocampi, left and right lenses). It will lead to poor conformity and homogeneity of the target in the slices containing these OARs. Whereas Fig. 6(b) showed that when the collimator is rotated to 90°, one MLC leaf pair is only responsible for sparing one side of the OAR (such as left hippocampus, left lens), thereby improving conformity and homogeneity of the target.

Results showed that the improvements above significantly reduced the dose to the hippocampus and enhanced the plan quality when compared with the conventional method. Meanwhile, the plans generated by the improved method were comparable to the HT plans, with slightly lower max hippocampal dose. Since the improved planning method was developed, the HA‐PCI based on VMAT technique has been massively used in our hospital, whereas the PCI without avoiding hippocampus using CRT has been deprecated. Up to now, nearly 100 patients have been treated with VMAT HA‐PCI. The max and mean doses to hippocampus received by all the patients were lower than 9 Gy and 7 Gy, respectively, which confirmed the reliability and stability of the improved method. Furthermore, this method also can be used for HA‐WBRT with metastases boost. Our practice indicated that the mean dose to hippocampus could be as low as 30% of PD, which might be a reference for other institutions.

Currently, research on head positioning for HA‐WBRT or HA‐PCI is limited. Siglin et al first studied the plans with various tilted angles for a head phantom. They found that when the phantom was tilted at a 30°, the max hippocampal dose was decreased by 34%. However, this result was inconsistent with that using data from real patients. Moon et al also compared the plans with 30° tilted head positioning to those with nontilted head positioning, and concluded that tilting patients’ head reduced the max hippocampal dose by an average of 16.2%. However, the comparison was performed among different patients and the number of subjects was small. In the present study, the tilt angle of 45° was chosen in order to rotate the patients’ eyeballs and nasal cavities out of the beam field, so as to provide maximum protection to normal tissue. Comparison showed that the max dose to lens and hippocampus were reduced by 37.5% and 7.1%, respectively, for the plans with tilted head positioning. Although the hippocampal reduction was not significant as previously reported, it can be observed from table 6 that the plans with tilted head positioning achieved a much more homogeneous dose distribution with much fewer MU. These results indicate that tilting patients’ head is beneficial for improving the plan quality. By placing a wedge with a 45° slope under the pillow, we realized 45° tilt of a patient’s head and the patient had no labored breathing or discomfort. However, the limitation of this study lies in using the rotated CT images to replace tilted head positioning, and the resulting difference was not clear. In the next study, we will put the tilted head positioning into clinical practice.

## 5 CONCLUSION

This study presented an improved VMAT planning method for HA‐PCI treatment. The plans generated using this method met the clinical requirements well, with a low dose to hippocampus and a high plan quality. Thus, we encourage to use this method instead of HT technique in clinical treatment. The tilted head positioning allowed to further reduce the dose to hippocampus and other normal tissue. We recommend viable institutions to use this positioning for HA‐PCI and HA‐WBRT.

## Conflict of Interest

No conflicts of interest.

## Author Contribution

Qi Fu, Yingjie Xu, Hui Yan: Conception and design of the study.

Qi Fu, Deqi Chen, Lei Deng: Treatment planning and collection of data.

Yingjie Xu, Lingling Yan, Jiayun Chen: Assessment of treatment plan.

Qi Fu, Deqi Chen, Ji Zhu: Analysis and interpretation of data.

Qi Fu, Yingjie Xu, Hui Yan, Jiayun Chen: Writing and revising the paper.

Yingjie Xu, Jianrong Dai: Final approval of the manuscript.
